# Minimal residual disease detection by next-generation sequencing in multiple myeloma: Promise and challenges for response-adapted therapy

**DOI:** 10.3389/fonc.2022.932852

**Published:** 2022-08-16

**Authors:** Valeria Ferla, Elena Antonini, Tommaso Perini, Francesca Farina, Serena Masottini, Simona Malato, Sarah Marktel, Maria Teresa Lupo Stanghellini, Cristina Tresoldi, Fabio Ciceri, Magda Marcatti

**Affiliations:** ^1^ Hematology and Bone Marrow Transplantation, San Raffaele Scientific Institute, Milan, Italy; ^2^ Molecular Hematology Laboratory, San Raffaele Scientific Institute, Milan, Italy; ^3^ Age Related Diseases Laboratory, Division of Genetics and Cell Biology, San Raffaele Scientific Institute, Milan, Italy; ^4^ University Vita-Salute San Raffaele, Milan, Italy

**Keywords:** multiple myeloma, next gene sequencing, treatment strategy, complete response, minimal residual disease

## Abstract

Assessment of minimal residual disease (MRD) is becoming a standard diagnostic tool for curable hematological malignancies such as chronic and acute myeloid leukemia. Multiple myeloma (MM) remains an incurable disease, as a major portion of patients even in complete response eventually relapse, suggesting that residual disease remains. Over the past decade, the treatment landscape of MM has radically changed with the introduction of new effective drugs and the availability of immunotherapy, including targeted antibodies and adoptive cell therapy. Therefore, conventional serological and morphological techniques have become suboptimal for the evaluation of depth of response. Recently, the International Myeloma Working Group (IMWG) introduced the definition of MRD negativity as the absence of clonal Plasma cells (PC) with a minimum sensitivity of <10^−5^ either by next-generation sequencing (NGS) using the LymphoSIGHT platform (Sequenta/Adaptative) or by next-generation flow cytometry (NGF) using EuroFlow approaches as the reference methods. While the definition of the LymphoSIGHT platform (Sequenta/Adaptive) as the standard method derives from its large use and validation in clinical studies on the prognostic value of NGS-based MRD, other commercially available options exist. Recently, the LymphoTrack assay has been evaluated in MM, demonstrating a sensitivity level of 10^−5^, hence qualifying as an alternative effective tool for MRD monitoring in MM. Here, we will review state-of-the-art methods for MRD assessment by NGS. We will summarize how MRD testing supports clinical trials as a useful tool in dynamic risk-adapted therapy. Finally, we will also discuss future promise and challenges of NGS-based MRD determination for clinical decision-making. In addition, we will present our real-life single-center experience with the commercially available NGS strategy LymphoTrack-MiSeq. Even with the limitation of a limited number of patients, our results confirm the LymphoTrack-MiSeq platform as a cost-effective, readily available, and standardized workflow with a sensitivity of 10^−5^. Our real-life data also confirm that achieving MRD negativity is an important prognostic factor in MM.

## Introduction

The treatment landscape for multiple myeloma (MM) has been radically changed during the past decade by the introduction of new drugs with different mechanisms of action, which has led to a significant improvement of survival (1).

Nearly all patients now achieve a treatment response, with more than half reaching a complete response (CR) that is defined by less than 5% plasma cells in the bone marrow (BM) regardless of their clonal nature and by the absence of monoclonal proteins in serum or urine by immunofixation or disappearance of soft tissue plasmacytomas. In 2006, the criteria of stringent complete response (sCR) were introduced (2), adding the normalization of the kappa and lambda serum free light chain (sFLC) ratio and the absence of clonal plasma cells in BM by immunohistochemistry (IHC) or immunofluorescence with a sensitivity of 10^−3^ (3, 4).

Despite achieving CRs, most patients eventually relapse, reflecting a persistent minimal residual disease (MRD) that cannot be detected with standard disease evaluation methods (5). Consequently, new techniques have been identified to detect a deeper response than CR; sensitive assays like next-generation sequencing (NGS) or eight-color next-generation flow cytometry (NGF) could further improve MRD detection. A third method, allele-specific oligonucleotide quantitative polymerase chain reaction (ASO-qPCR), has also been extensively studied, but the need for patient-specific primers, high technical complexity, and low applicability limit its use.

In 2016, the IMWG established new consensus criteria to define MM disease response that include MRD assessment. MRD should be assessed when a patient achieves a CR or better, with a minimum sensitivity of one nucleated tumor cell in 100,000 normal cells (10^-5^ sensitivity), by either NGS or NGF (5, 6).

MM is a heterogeneous and patchy disease within the BM; these disease characteristics need to be considered in MRD evaluation (7). Additionally, the presence of extramedullary disease (EMD) may lead to false-negative MRD results (8, 9). These limitations, together with patients’ discomfort with BM evaluation, have led to efforts to individuate alternative approaches to test MRD.

In particular, positron emission tomography–computed tomography (PET-CT) can have a complementary role to marrow MRD assessment. The absence of metabolically active areas after treatment had already been correlated with improved clinical outcomes. Moreover, it proved to be an independent outcome predictor (10). For example, the CASSIOPEA study has identified a concordance of 61.9% between MRD and PET-CT negativity post consolidation, with 6.8% of patients showing PET-CT positivity and MRD negativity (11). These observations demonstrate the failure of BM-based assays to detect MRD in specific cases (7, 11, 12). Indeed, PET-CT has already been included as a separate subcategory in the latest IMWG response criteria (5).

Liquid biopsy has also been proposed as an alternative approach for tracking MRD after treatment based on the hypothesis that a peripheral blood sample would be able to reflect BM and/or EMD status. Current approaches for liquid biopsy testing are mostly based on circulating tumor DNA (ctDNA). While one study (13) reported that ctDNA levels were significantly correlated with MRD levels obtained by 8-color flow cytometry, other studies reported detectable ctDNA in less than 50% of patients with very good partial response (VGPR) or worse response after treatment (14, 15) or revealed a low correlation between ctDNA and flow-MRD status (16). Even if a possible explanation for low concordance is that ctDNA may decline more rapidly than residual disease in other compartments after effective treatment (14, 17, 18), these assays are still relatively new and with several open issues, thus not constituting the currently preferable choice for MRD evaluation.

Currently, MRD has been incorporated in numerous clinical trials with different aims, as follows: 1) as a prognostic factor; 2) as a surrogate end to evaluate treatment efficacy and compare different treatment approaches; 3) as an adapted treatment strategy according to MRD status and its follow-up especially in maintenance duration and early salvage approach.

## Minimal residual disease as a prognostic factor

The association between depth of response and long-term outcome is a current topic of debate in MM. CR has been generally considered as the best possible response after treatment and an indicator of improved outcome. The value of sCR compared to CR is still controversial, with only one out of four studies reporting a better prognosis after autologous stem cell transplantation (ASCT) when sCR rather than CR was achieved (19–22).

On the opposite, MRD is emerging as a more sensitive assessment of depth of response and a more powerful prognostic tool, with many studies demonstrating that undetectable MRD is associated with improved progression-free survival (PFS) and overall survival (OS) (23–31).

The strongest evidence, so far, comes from a large meta-analysis of MRD data of 44 studies for PFS and 23 studies for OS. Achieving MRD negativity led to improved PFS and OS regardless of several disease and patient characteristics. The beneficial effect of MRD negativity was sustained regardless of MRD sensitivity thresholds (older studies reported results with a sensitivity of 10^-4^), cytogenetic risk, method of MRD assessment, and depth of clinical response at the time of MRD measurement (26).

Strong evidence also comes from a pooled analysis of four phase III clinical studies of daratumumab plus standard-of-care regimens in relapsed patients and in transplant-ineligible newly diagnosed disease (ALCYONE, CASTOR, MAIA, and POLLUX) that demonstrates the high efficacy of daratumumab-based regimens in improving MRD negativity rates and reducing the risk of disease progression or death. In all of these studies, MRD was assessed by NGS using the clonoSEQ assay (v.2.0; Adaptive Biotechnologies, Seattle, WA, USA) with a sensitivity of 10^-5^, and patients who achieved CR or more with MRD negativity had improved PFS compared to those who failed to reach CR and were MRD positive. This large-scale analysis examined a homogeneous population using similar intervals of periodic MRD assessments applying uniform MRD assessment techniques and thresholds, supporting MRD negativity as a prognostic tool for PFS in transplant-ineligible newly diagnosed multiple myeloma (NDMM) and relapsed or refractory multiple myeloma (RRMM) (32).

Another example supporting the value of MRD in prolonged survival is the phase III PETHEMA/GEM2012MENOS65 trial. MRD was assessed by NGF in BM samples in NDMM after bortezomib-lenalidomide and dexamethasone (RVD) induction, autotransplant, and two RVD consolidation cycles in order to modify accordingly the maintenance therapy. Importantly, with a median follow-up of 40 months, patients with undetectable MRD after consolidation showed very low risk of disease progression (7%), with a 3-year survival rate reaching 90%. Attaining undetectable MRD also overcame poor prognostic features at diagnosis, including high-risk cytogenetics, thus confirming MRD as the strong prognostic factor for MM (33).

Several other studies support these conclusions and confirm their value in different patient populations at different time points and with several MRD assessment methods.

In conclusion, MRD negativity is associated with improved survival independently of the disease status (newly diagnosed or relapsed disease), cytogenetic risk, MRD sensitivity level, method used for MRD assessment, and the level of the clinical response at the time of MRD evaluation negativity. Moreover, MRD negativity is the most relevant predictor of clinical outcome compared with other prognostic factors (23–26, 28, 30, 31, 34–38).

## State of the art of next-generation sequencing for minimal residual disease assessment

The IMWG defined MRD negativity as the absence of clonal plasma cells by either sequencing- or flow cytometry-based techniques with a minimum sensitivity of <10^−5^ using as the reference methods (5) the LymphoSIGHT (Sequenta/Adaptative) (24) and NGF EuroFlow approaches (39). IMWG does not favor either NGF or NGS technology because the defined 10^−5^ sensitivity is obtainable with both.

Multiparametric flow cytometry (MFC) allows highly sensitive discrimination between polyclonal and clonal plasma cells based on their immunophenotypic characteristics (normal vs. aberrant). MFC detects cell surface markers using fluorescently labeled antibodies. Canonical markers include CD138, CD38, CD45, CD56, CD19, and cytoplasmic κ and λ immunoglobulin light chain, while other markers include CD20, CD27, CD28, CD81, CD117, and CD200. In order to overcome the limitations of conventional MFC, highly sensitive NGF MRD approaches have been developed in the last years by EuroFlow. Current NGF methods rely on a more efficient sample preparation protocol that allows acquisition of >10 million BM cells (eight-color two-tube panel) and on innovative automatic data analysis software tools that have resulted in increased sensitivity of 10^-6^ (5, 33, 39, 40).

NGS consists of the molecular assessment of immunoglobulin gene rearrangements, and it allows to detect MM MRD in the BM. The development of NGS methods for MRD assessment has focused mainly on the third complementarity determining region (CDR3), a sequence of 30–70 nucleotides, at the intersection of variable (V), diversity (D), and joining (J) immunoglobulin gene segments (41). During formation of the mature immunoglobulin gene, vast diversity is introduced through V(D)J recombination, junctional insertions/deletions, and somatic hypermutation. Therefore, this rearrangement pattern is patient-specific and provides a cancer cell-specific measure. NGS uses primers to amplify gene fragments and then sequences immunoglobulin gene segments within IgH (VDJ), IgH (DJ), IgK, and IgL receptor gene sequences.

Once amplified, the immunoglobulin gene DNA is sequenced and the frequencies of the different clonotypes are determined. To avoid disproportional amplification of the immunoglobulin heavy chain gene (IGH) rearrangements and immunoglobulin light chain gene (IGK) rearrangements, the extensive sets of primers need to be attuned and validated to guarantee correct and proportional amplification of each target rearrangement compared with many rearrangements derived from normal B cells (42).

These gene rearrangements must be studied at diagnosis in a baseline sample; baseline clonality detection rates vary between 80% and 97% in different studies using different NGS platforms (24, 28, 31, 35, 39, 43). As MM is a post-germinal center B-cell disease, IGHV and IGK rearrangements often bear an extensive imprint of somatic hypermutation (SHM). This is in contrast to, for example, B-lymphoblastic leukemia, where the neoplastic cells have not yet undergone SHM. Of note, lambda-restricted MM has a higher probability clonality detection because clonal IGK rearrangements are identified more frequently in this group and there are fewer SHMs of IGK clonal rearrangement sequences in lambda-restricted cases than kappa ones.

Extensive SHM can interfere with PCR primer annealing, resulting in less effective amplification of V(D)J rearranged sequence and making the clonal sequence indistinguishable from the background of normal polyclonal B cells. When using primers that anneal inside the IGHV exon (FR1, FR2, FR3), the myeloma-associated rearrangement may be missed, making impossible to determine clonality. This could be avoided by using primers that bind in the leader region targeting the leader sequences upstream of the VH region avoiding amplification bias due to SHM. However, these approaches lack validation data for MRD tracking (44–46).

The NGF and NGS technology platforms have different advantages and limitations; **Table 1** compares some of the salient features of each of these techniques.

**Table 1:** Comparison of NGS and NFG for assessment of MRD in multiple myeloma.

	NGS	NGF
Applicability	>90%	Nearly 100%
Baseline sample required	Baseline sample required for identification of the dominant clonotype	Not required
Method	Specific immunoglobulin rearrangements are identified and detected by comparison with baseline sample	Abnormal (clonal) plasma cells are identified by their distinct immunophenotypic pattern vs. normal plasma cells
Turnaround time including analysis time	>7 days	5 hours
Sensibility	10^-6^	10^-6^
Quantity of sample required	3 million for 10^-6^ sensitivity; higher numbers improve sensibility	Up to 10 million for 10^-6^ sensitivity
Clonal evolution	Evaluable	Not evaluable
Standardization	Commercial companies	EuroFlow consortium
Sample processing	Can be delayed; both fresh and stored samples can be used	Needs assessment within 24–48 h; requires a fresh sample
Cost sample	++/+++	+
Sample quality control	Not possible	Immediate with bone marrow analysis

NGF has an analytical sensitivity of 2 tumor cells in 1 million (10^−6^) BM cells if 10 million cells can be acquired; NGS has an analytical sensitivity of 10^−6^ once a DNA input from 3 million BM cells is granted. Therefore, NGS requires a smaller specimen volume than NGF. Moreover, NGS can be applied retrospectively on stored material including not only cryopreserved cells but also archival BM slides, while NGF requires a fresh sample that has to be processed within 24 h from aspiration.

NGS requires a baseline patient sample for identification and detection of MRD, while NGF can detect abnormal cells in any given sample. NGS analysis is performed in specialized laboratories and thus requires the specimen to be shipped for processing, often resulting in a longer turnaround time (approximately 7 days) than NGF (24–48 h).

A novel, not yet clinically approved, NGS approach, the LymphoTrack-MiSeq platform, potentially overcomes this pitfall because it could potentially be performed at individual institutions, with a higher degree of patient applicability. Currently, the turnaround time of the LymphoTrack-MiSeq platform for fresh samples is about 5 days [Day 1: sample processing, DNA extraction, and preparation and purification of the libraries; Day 2: kappa library (for quantification of libraries) and loading of pooled libraries on MiSeq; Days 3–4: run on MiSeq; Day 5: data analysis].

NGS is still an expensive technique, with the cost per sample about twice or thrice that of NGF; however, prices vary for different platforms, and the LymphoTrack-MiSeq platform is among the least expensive and can be competitive with NGF. Moreover, NGS costs can be reduced by testing multiple samples at the same time.

Most published data have been generated with the LymphoSIGHT platform (Sequenta Inc., San Francisco, CA, USA). Notably, the sensitivity threshold was claimed to be of at least 10^−5^ (24, 31) or 10^−6^ (35, 47, 48), depending on the different studies. The 10^−7^ sensitivity has also been reached but requires high amounts of DNA that make it infeasible in clinical practice (49). Another NGS-based platform, the clonoSEQ Assay (Adaptive Biotechnologies, Seattle, WA, USA), obtained clearance from the US Food and Drug Administration in 2019 for the detection and monitoring of MRD in BM samples from MM patients or B-cell acute lymphoblastic leukemia. The European Medicines Agency (EMA) has issued guidance on the use of MRD in MM studies but has not adjudicated the use of this or any other method.

More recently, the LymphoTrack-MiSeq platform, a commercial strategy designed by *In Vivo*Scribe Technologies, has been evaluated for MRD detection in MM patients demonstrating a minimum sensitivity level of 10^−5^, hence qualifying as an alternative effective tool for MRD monitoring in MM (50, 51).

Several clinical studies investigated the prognostic value of NGS technology in MM management and compared it with the other MRD tools.

Compared to ASO-qPCR, NGS has similar sensitivity (at least 10^−5^) but has the advantage of not requiring patient-specific primers (52).

Martinez-Lopez et al. (24) compared MRD using the LymphoSIGHT sequencing platform with MFC and ASO-qPCR. The applicability of deep sequencing was very high (91%). Concordance between sequencing and MFC and ASO-qPCR was 83% and 85%, respectively (24).

In the IFM2009 (bortezomib, lenalidomide, and dexamethasone plus up-front vs. deferred transplant), MRD was originally evaluated by first-generation flow cytometry (seven-color MFC), showing that patients who were MRD-negative with a sensitivity of 10^-4^ had improved PFS. As NGS techniques became more widely available, stored samples were reapproached using the LymphoSight platform (Sequenta/Adaptive Inc.), reaching a sensitivity of 10^-6^. This follow-up study demonstrated that the ability to measure deeper response provides superior outcome. With 10^−6^ sensitivity, MRD negativity was a strong prognostic biomarker of PFS and OS. This approach demonstrated a higher level of discrimination than had previously been achieved with the less sensitive MFC technique (28, 53, 54).

The concordance between MRD evaluation by NGS and MFC was analyzed in the FORTE trial. MRD was assessed by NGS or by eight-color second-generation flow cytometry (sensitivity 10^−5^) and by NGF in subgroup patients. MFC and NGS evaluations with high sensitivity (sensitivity 10^−5^–10^−6^) were available only in ≥CR patients, while MRD was analyzed both by NGS and second-generation MFC in patients achieving VGPR or better. In 30% of cases, a discordance between NGS and second-generation MFC was detected: in all of the discordant cases but one, MRD positivity was missed by MFC. However, when NGF was compared with NGS in the subgroup of CR patients, results were highly concordant with the two techniques (83%) (55).

Even in the CASSIOPEIA trial, MRD has been evaluated with both NGS and NGF. A good concordance (83.5%) was observed using the same sensitivity threshold (10^−5^), regardless of response in patients achieving CR or more, indicating that both techniques performed similarly in evaluating MRD (56–58).

Another study analyzed patients from the GEM2012 trial, comparing the NGF (Euroflow) and NGS (LymphoTrack) methods for MRD detection. NGS showed good concordance with NGF, with only 15 out of the 106 patients studied having contradictory results. Most discordant cases had MRD levels below 10^−5^ that either may be explained by a higher sensitivity for one method over the other or can be explained with technical inaccuracies or in differences in the sampling procedure (51).

In conclusion, the NGS approach is a powerful tool for MRD detection, considering the key role of the achievement of MRD negativity in the clinical management of MM patients. During the last few years, several NGS platforms for MRD detection in MM were tested but there are no data that compare the different platforms yet.

## Single-center experience with the LymphoTrack-MiSeq platform

We present our single-center experience with the LymphoTrack-MiSeq platform for the diagnosis and MRD detection in a selected cohort of MM patients. Our aim was to test the applicability and verify a sensitivity of at least 10^−5^ of a new commercial NGS panel.

### Patients and samples

In 2018, we retrospectively selected 28 consecutive transplant-eligible newly diagnosed MM patients. We selected 52 samples, consisting of 28 diagnoses and 24 follow-up specimens at day +100 following ASCT. BM aspirates were collected at diagnosis and subsequently at follow-up from our Institutional Biobank. All patients included in the present study provided a written informed consent to have their biological samples stored and characterized.

### DNA sample quality check and quantification

Genomic DNA (gDNA) was isolated from BM aspirates with the automated Maxwell 16 system using 16 LEV Blood DNA kit according to the manufacturer’s instructions (Promega, Madison, WI, USA). gDNA was isolated from total white blood cells (WBC) after lysis of red blood cells. In some cases, DNA was extracted immediately after sample processing according to Maxwell manufacturer’s protocol. In other cases, cells were resuspended with a solution of 10% Dimethyl Sulfoxide (DMSO) and 90% fetal bovine serum (FBS) and stored in liquid nitrogen. After the extraction, gDNA was conserved at -20°C. For long-term storage (over 1 year), DNA was conserved at –80°C.

After the extraction, gDNA purity was checked using the Nanodrop spectrophotometer (Thermo Scientific, Wilmington, MA, USA) considering absorbance ratio at both 260/280 and 230/260 nm. In order to verify gDNA quality, we analyzed its size distribution electrophoresis on 1% agarose gel. Samples with smears below 2.5 kb were classified as degraded and were excluded from further analysis. DNA concentration was measured by the Qubit dsDNA HS assay kit and Qubit 2.0 fluorometer (Life Technologies, Grand Island, NY, USA).

### Library preparation and sequencing

Library preparation was performed using the LymphoTrack Dx IGH (FR1, FR2, FR3) and IGK assays according to the manufacturer’s recommendation (*InVivo*Scribe Technologies, San Diego, CA, USA). Briefly, LymphoTrack Dx IGH (FR1, FR2 and FR3) and IGK Assays include a single multiplex master mix that targets one of the conserved IGH framework regions (FR1, FR2, or FR3) within the VH and JH regions and Vk-Jk, Vk-Kde, and INTR-Kde gene rearrangements, respectively. Each master mix contains primers with Illumina adapters and up to 24 different indices that allow library preparation in one-step PCR reaction. Amplicons were purified using AMPure XP beads (Beckman Coulter, Brea, CA, USA) and quantified with KAPA Library Quantification Kit for Illumina platforms (Roche, Basel, Switzerland). Here, 4 nM of each library was pooled, denatured, and diluted to 12–20 pM. Sequencing was performed in the MiSeq platform (Illumina, San Diego, CA, USA) using v3 (600 cycles) reagent kits. FASTQ files generated from sequencing were analyzed using the LymphoTrack-Miseq and LymphoTrack MRD Software (*InVivo*Scribe Technologies, San Diego, CA, USA).

For the 28 samples of newly diagnosed patients, clonality was assessed by both LymphoTrack IGH (FR1, FR2, or FR3) and IGK assays. Each MRD sample was analyzed in triplicate, and for each replicate, 500–1,500 ng of DNA was used as input (total DNA analyzed 1,500–4,500 ng); for each replicate, we obtained at least 1,080,000 total reads. Experiment setting up was performed using Lymphotrack MRD software according to the manufacturer brochure available at the time (2020) that indicated a minimum of 1,000 ng per replicate to reach 10^-5^ sensitivity with 95% confidence. Based on those requirements, some of our samples fell below this threshold because of insufficient input DNA (<1,000 ng per replicate). Importantly, 1,000 ng per replicate was loaded for all of the MRD-negative samples (6/24, see below), ensuring 10^-5^ sensitivity. Manufacturer’s brochure was updated in 2021 and now reports a minimum requirement of 2,000 ng total gDNA analyzed and a total read depth of 44 million reads to reach 10^-5^ sensitivity with 95% confidence of a true MRD negativity. This real-life experience reveals how standardization and optimization of NGS techniques are still ongoing and crucial.

### Results and minimal residual disease assessment by next-generation sequencing

We retrospectively selected 28 consecutive transplant-eligible newly diagnosed MM patients (12 men and 16 women; median age at diagnosis 55 years, range 17–71 years). All patients received VTd induction (bortezomib–thalidomide–dexamethasone) and tandem ASCT. No maintenance therapy was used. Clinical variables of our cohort are described in **Table 2**, including ISS and R-ISS prognostic score, cytogenetic risk assessed by FISH for del17p, t (4;14), t (14;16), BM infiltration, and bone lesions.

**Table 2:** Patient characteristics.

Characteristics	N = 28
Sex, men/women	12/16
Age, median (range), years	55 (17–71)
Type of multiple myeloma (IgG/non-IgG)	18/28
Bone marrow plasma cells >60%	4/28
Bone lesions Monofocal Multifocal	23/28 2/23 21/23
Cytogenetic profile	
Standard risk high risk, del 17 p t (4;14)	17 2 1/2 1/2
ISS	
I II III	17/28 7/28 3/28
Response to therapy at 100 days following first ASCT	
sCR CR VGPR PR	13/28 1/28 12/28 2/28
Disease progression	18/28
Overall survival, months (range)	77 (25–145)
Dead	4/28

We evaluated clonal rearrangements of IGH (FR1, FR2, FR3) and IGK target region by the LymphoTrack-MiSeq platform on BM samples at two time points: at diagnosis and at day 100 following first ASCT. At diagnosis, 26 samples (26/28) showed monoclonality and 2 samples (2/28) polyclonality. Interestingly, these two patients had EMD at PET/CT evaluation. In the evaluation of the single targets (IgH and IgK), the results highlighted the presence of monoclonality for the IgH loci in 7 samples (7/26) and for the IgK loci in 4 samples (4/26) while 15 samples (15/26) in both of them. At day 100 after ASCT I consider only 24 patients who had evaluable MDR. We evaluated MRD at day 100 after first ASCT: 2 patient samples were not evaluable, and MRD was positive in 18 patients (18/24) and negative in 6 (6/24) patients. In the evaluation of the single targets (IgH and IgK) at day 100 post first ASCT, the results highlighted the presence of monoclonality for the IgH loci in 7 samples (7/24) and for the IgK loci in 4 samples (4/24), in 7 samples (7/24) for both of them. Such variations are in line with disease heterogeneity in which the dominant clone is eradicated during treatment, but resistant and quiescent subclones persist and inevitably proliferate, resulting in relapsing disease.

The response to treatment was evaluated according to IMWG criteria at different time points: after induction (5 sCR, 4 CR, 17 VGPR, 2 PR) and at day 100 following first ASCT (13 sCR, 1 CR, 12 VGPR, 2 PR). Among patients who reached sCR at day 100 following first ASCT, five patients were MRD-negative and six patients were MRD-positive; a single MRD-negative patient was in CR and 10 patients in VGPR were MRD-positive and one patient was MRD-negative.

At a median follow-up of 81 months (range: 25–149 months), 15 out of 24 MRD evaluated patients relapsed during follow-up, 2 in the MRD-negative group (2/6) and 13 in the MRD-positive group (13/18). The time to next treatment was 87 months for MRD-negative patients (range 35–140 months), while the median time to next treatment was significantly low in the MRD-positive patients: 44 months (range 14–90 months; log rank test: P = 0.175).

At the last follow-up, five patients in the MRD-positive group had died (5/28), one due to infection and four for progressive disease. In the MRD-negative group, all patients are alive. Among the MRD-negative patients, four out of six are maintaining complete remission until the last control.

**Figure 1:**
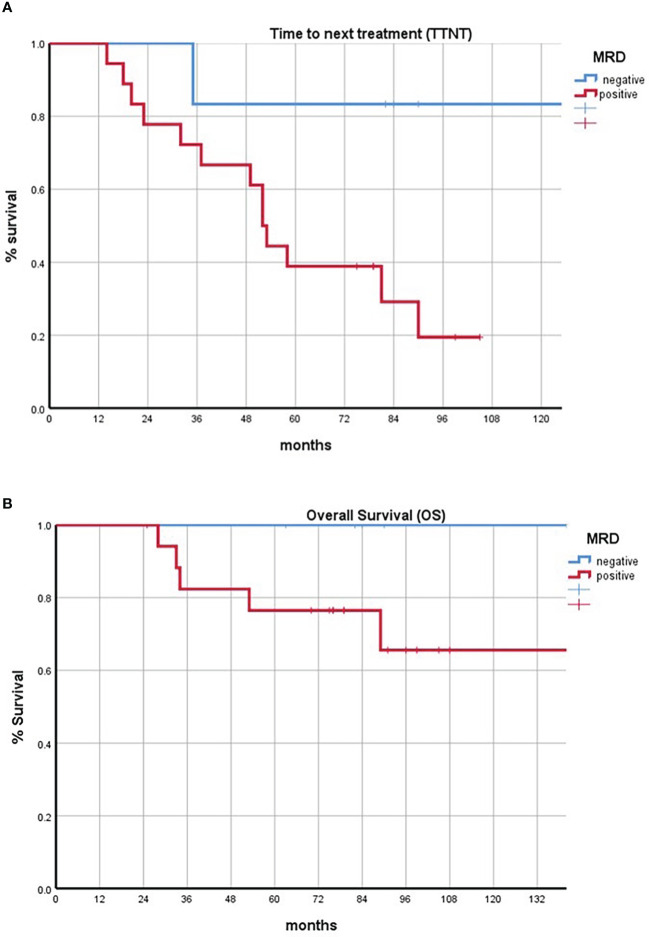
**(A)** Kaplan–Meier curves comparing the time to next treatment of minimal residual disease (MRD)-positive and MRD-negative subsets. **(B)** Kaplan–Meier curves comparing the overall survival of MRD-positive and MRD-negative subsets. Negative patients are represented in blue; positive patients are represented in red.

The OS of all our cohorts from diagnosis was 82 months (range: 25–150 months), with 23 patients still alive. The OS was 90 months for MRD-negative patients (range 63–140), while the median OS in the MRD-positive group was 71 months (range 25–150 months; log rank test: P = 0.038) (**Figures 1A, B**).

Our pilot experience with LymphoTrack is in line with the other studies that support the prognostic role of MRD. Moreover, we tested the feasibility of this platform: this assay offers a sensitive and precise method for diagnostic testing and disease MRD monitoring. In particular, the LymphoTrack bioinformatics software allows a simple automated visualization of data and analysis, demonstrating a consistent and reliable routine clinical testing.

## Minimal residual disease as an end point in clinical trials

Therapeutic advances have greatly increased outcome in MM patients. However, the usage of classical end points such as OS or PFS needs longer follow-up. Given the stringent correlation of MRD negativity with prognostic parameters, MRD evaluation has been widely accepted as a robust surrogate method for measuring the clinical end points of current clinical trials. Indeed, MRD can also be used as a marker of efficacy in new combination therapy for accelerated drug development and fast approval. For example, the phase III CASSIOPEIA trial demonstrates the clinical benefit of daratumumab plus VTd (D-VTd) in transplant-eligible patients with newly diagnosed MM. The MRD was assessed by MFC during induction/consolidation and additionally by NGS during maintenance, both with the 10^–5^ sensitivity. MRD status is reported for patients who achieved a CR or better. During induction and consolidation therapy, D-VTd leads to increased rates of MRD negativity and prolonged PFS associated with sustained MRD negativity at 1 and 2 years of follow-up. Secondly, daratumumab maintenance is compared to observation. Daratumumab before and after ASCT significantly improves PFS and depth of response, as demonstrated by significant increases in MRD-negative rates vs. VTd. Sustained MRD negativity was higher in the D-VTd group compared to VTd at 1 year (50.1% vs. 30.1%) and at 2 years (35.5% vs. 18.8%) (56–58).

An MRD correlative study conducted in a randomized phase 3 study (EMN02 HOVON95) supported the role of lenalidomide maintenance in patients with newly diagnosed MM after different induction strategies. In patients achieving a CR before maintenance BM samples were processed, applying EuroFlow-based MFC protocols (eight colors, two tubes, with 10^-4^–10^-5^ sensitivity), after a median follow-up of 75 months, 5-year PFS was 66% in MRD-negative patients vs. 31% in MRD-positive patients, and 5-year OS was 86% vs. 69%, respectively. In the 1-year maintenance MRD population, 42% of MRD-positive patients at pre-maintenance became MRD-negative after lenalidomide exposure. The authors concluded that MRD by MFC is a strong prognostic factor and that lenalidomide maintenance can improve MRD negativity rate and therefore prolong OS (59).

Another example is the MANHATTAN trial, a non-randomized phase II trial that evaluates the addition of daratumumab to the combination of carfilzomib, lenalidomide, and dexamethasone (KRD) in patients with newly diagnosed MM. The primary end point was the MRD (MRD <10^-5^ sensitivity) rate in the absence ASCT. MRD negativity was achieved in 71% of patients. Median time to MRD negativity was 6 cycles. At 11 months of the median follow-up, the 1-year PFS rate and the OS rate were 98% and 100%, respectively. This rate of MRD negativity allowed the authors to underline the efficacy in response rates and PFS of the new combination therapy based on carfilzomib-lenalidomide-dexamethasone-daratumumab (27).

## Minimal residual disease study-adapted strategy

Given its power in predicting long-term outcomes, MRD represents an attractive tool to potentially guide treatment choices.

Phase II/III clinical trials can explore treatment intensification or deintensification according to MRD status after treatment at different time points or during maintenance. MRD in the different studies is valuated with NGS (different commercial platforms) or NGF (threshold of 10^-5^).

For example, a Minimal Residual Disease Adapted Strategy (MIDAS) (NCT04934475) is an interventional and prospective phase III randomized trial. After an induction regimen consisting of isatuximab, carfilzomib, lenalidomide, and dexamethasone (Isa-KRD), MRD status is determined by NGS and used to classify patients as standard (MRD <10^-5^) or high (MRD >10^-5^) risk. Standard-risk patients are randomized to six additional cycles of Isa-KRD or ASCT followed by two cycles of Isa-KRD, while high-risk patients are randomly assigned to ASCT followed by two cycles of Isa-KRD or tandem ASCT. Also, maintenance is informed by post-induction MRD status, as standard-risk patients receive lenalidomide for 3 years and high-risk patients receive iberdomide and isatuximab for 3 years (57). Results are still awaited.

The MASTER trial (NCT03224507) is a multicenter, single-arm, phase II study in which NDMM patients received induction with daratumumab, carfilzomib, lenalidomide, and dexamethasone (Dara-KRd) followed by autologous transplant and Dara-KRd consolidation. MRD evaluation by ClonoSEQ NGS (MRD <10^-5^) was used to inform therapy at two time points. First, Dara-KRd consolidation was interrupted in patients with two consecutive MRD-negative assessments. Second, patients who completed consolidation therapy and remained MRD-positive received lenalidomide maintenance. This study demonstrated how MRD response-adapted consolidation leads to higher rates of MRD negativity a higher PFS in NDMM, offering an alternative strategy to indefinite maintenance (1).

In the recruiting phase 3 DRAMMATIC (NCT04071457) study, after induction and ASCT, patients are randomized between lenalidomide and lenalidomide with daratumumab/rHuPH20. After 2 years of maintenance, MRD is assessed to guide further therapy. MRD-positive patients will continue with the assigned treatment. MRD-negative patients will be further randomized to continue/discontinue the assigned treatment.

In the recruiting phase 2–3 study REMNANT (NCT04513639), newly diagnosed patients are treated with standard induction (RVD), ASCT (single or tandem), and consolidation. Patients who reach MRD negativity (<10^-5^, Euroflow NGF) post consolidation are randomized to receive second-line treatment at MRD reappearance (arm A) or at progressive disease as defined by the IMWG criteria (arm B) in order to evaluate whether treating MRD relapse after first-line treatment prolongs PFS and OS for myeloma patients vs. treating relapse after progressive disease.

A recruiting phase 2 single-arm clinical trial (NCT04140162) will test MRD status after induction therapy with DRd combination (daratumumab, lenalidomide, and dexamethasone): MRD-positive patients will receive consolidation therapy with DRVd (daratumumab, lenalidomide, bortezomib, and dexamethasone), with the aim of testing if MRD-adapted consolidation will result in more patients achieving MRD negativity.

In the phase 3 AURIGA (NCT03901963) study, patients who remain MRD-positive after frontline ASCT are randomized to daratumumab-lenalidomide or lenalidomide alone in order to evaluate the intensification of maintenance therapy with daratumumab. NGS is used to assess the conversion rate to MRD negativity during maintenance.

A phase 2 recruiting study, DART4MM (NCT03992170), is now evaluating the effect of daratumumab administration in patients who test MRD-positive by NGF after any therapy (ASCT, VMP, Rev-Dex). If patients will be still MRD-positive after 6 months of therapy, treatment will be continued up to 2 years. If MRD negativity will be reached, daratumumab will be suspended.

All these trials are examples of how the spectrum of uses of MRD in the future will expand and become increasingly crucial in everyday choices.

## Minimal residual disease in therapeutic decision: Ready for minimal residual disease-guided therapy?

**Figure 2:**
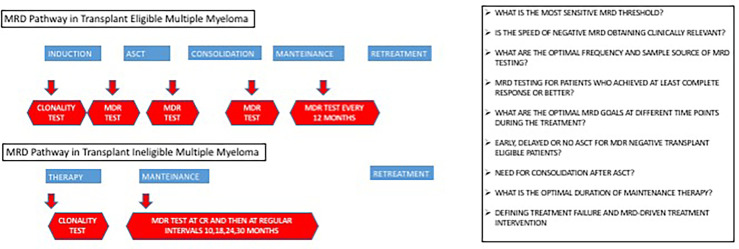
Future minimal residual disease (MRD) prospects in a multiple myeloma clinical trial.

MRD detection is widely used in several hematological malignancies (e.g., myeloid chronic leukemia, acute lymphoblastic leukemia), both for risk assessment and therapeutic choices. In MM, the prognostic value of MRD has been amply demonstrated, but its role in therapeutic decision is still a matter of debate (1, 60–67). Indeed, while ongoing clinical trials are studying the role of MRD status in clinical management (**Figure 2**), many questions remain open.

A crucial discussion regards the identification of optimal time points of MRD testing. Now, no standard rule exists, and the matter is further complicated by the possible additional value of repeated MRD measurement over time rather than a single determination at the first achievement of CR.

Many studies are exploring the role of MRD testing in defining the timing of ASCT. In particular, the question is whether patients who achieve deep MRD-negative responses after induction therapy should harvest stem cells but delay ASCT until salvage therapy after disease progression (33, 54, 55, 63). Similarly, MRD could be a useful tool to define the need for consolidation therapy after ASCT (68).

Another possible application of MRD is in the definition of the optimal length of maintenance therapy following ASCT. Current data suggest an advantage of continuous maintenance therapy (69–72), but cost-of-care and toxicity implications due to long-term maintenance should be taken into consideration. Of note, many ongoing clinical trials are testing the efficacy of maintenance cessation upon achievement of sustained MRD negativity. In a similar approach, several trials are randomizing MRD-positive patients to continuous maintenance or switching therapy (63).

Similar questions exist also for the non-transplant-eligible patients, where the positive impact of continuous treatment on OS is not well demonstrated (71, 73, 74). Clinical trials where MRD-negative patients are randomized to therapy suspension or continuation until disease progression or intolerance will prove informative.

Similar to other hematological malignancies, MRD could also be used to guide retreatment. The current paradigm for relapsing patients is a relative delay of retreatment initiation until appearance of CRAB (hypercalcemia, renal insufficiency, anemia, or bone lesions) features (5, 71). Though many pieces of evidence support the role of MRD to define treatment failure, to our knowledge, only one ongoing trial is designed to explore the potential benefit of an early treatment intervention based only on the reversal of the MRD status from negative to positive (NCT04513639); interim analysis is ongoing.

## Conclusions

In this work, we summarize and comment on the status of MRD testing in MM. In particular, our review retraces the recent and ongoing clinical trials that demonstrate or explore the prognostic role of the different MRD testing techniques. We also summarize the current efforts to determine the power of MRD as a tool in dynamic risk-adapted therapy.

We also test the potential applicability and usefulness of a new commercial NGS panel, the LymphoTrack-MiSeq NGS methodology, for the diagnosis and MRD detection in our court of MM patients. Our work highlights the advantages of NGS over flow cytometry for MRD testing. One potential perceived disadvantage of the NGS test is that it is performed in a central laboratory, and results require more time as compared with in-house tests. Our single-center pilot experience supports the potential applicability of a commercial NGS panel and the value of MRD determination by the LymphoTrack-MiSeq platform as a cost-effective, readily available, and sensitive workflow. Even if the limit of our analysis is the low number of patients and the sensitivity level of the method adopted (acceptable in 2018), our results are in line with all available studies that confirm that achieving MRD negativity is an important prognostic factor in MM.

While the high efficacy of new treatment strategies requires highly sensitive tools to monitor disease status and inform clinical decisions, the role of MRD testing in MM is currently limited to clinical trials. With the increasing validation of its prognostic role, MRD testing will move from passive (i.e., a measure of the extent of treatment effectiveness) to active (i.e., a tool to guide treatment choices) and will leave the clinical trial setting to be included in standard care. For these reasons, NGS standardization is of extreme importance for the clinical applicability of MRD detection. While NGS can have discrepant results because of interlaboratory differences, variable performance of the available technologies, and site-specific sample collection procedures, strict standardization will ensure consistent and comparable results. Ongoing efforts are aimed at reaching uniform timing of BM collection and sample processing and at defining the amount of DNA input, number of replicates, number of sequencing reads, and sequence similarity thresholds for determining clonotypes. With the aim of implementing MRD in MM everyday patients’ care, careful standardization, consistent sensitivity, reproducibility, and affordability of NGS-MRD assays will be fundamental (1, 75, 76).

## Author contributions

FV, EA, CT, TP, MM designed the study. collected study data. EA, SeM, CT performed laboratory analysis. FV, EA, TP, FF wrote the manuscript draft. MM, MTLP, FC edited the manuscript draft and did subsequent revision. All authors listed have made a substantial, direct, and intellectual contribution to the work, and approved it for publication.

## Conflict of interest

The authors declare that the research was conducted in the absence of any commercial or financial relationships that could be construed as a potential conflict of interest.

## Publisher’s note

All claims expressed in this article are solely those of the authors and do not necessarily represent those of their affiliated organizations, or those of the publisher, the editors and the reviewers. Any product that may be evaluated in this article, or claim that may be made by its manufacturer, is not guaranteed or endorsed by the publisher.

## References

[1] Costa LJ Derman BA Bal S Sidana S Chhabra S Silbermann R. International harmonization in performing and reporting minimal residual disease assessment in multiple myeloma trials. Leukemia (2021) 35(1):18–30. doi: 10.1038/s41375-020-01012-4 32778736

[2] Durie BG Harousseau JL Miguel JS Bladé J Barlogie B Anderson K. International uniform response criteria for multiple myeloma. Leukemia (2006) 20(9):1467–73. doi: 10.1038/sj.leu.2404284 16855634

[3] Chee CE Kumar S Larson DR Kyle RA Dispenzieri A Gertz MA. The importance of bone marrow examination in determining complete response to therapy in patients with multiple myeloma. Blood (2009) 114(13):2617–8. doi: 10.1182/blood-2009-01-198788 PMC2756122 19641191

[4] Matsue K Matsue Y Kumata K Usui Y Suehara Y Fukumoto K. Quantification of bone marrow plasma cell infiltration in multiple myeloma: usefulness of bone marrow aspirate clot with CD138 immunohistochemistry. Hematol Oncol (2017) 35(3):323–8. doi: 10.1002/hon.2300 27140172

[5] Kumar S Paiva B Anderson KC Durie B Landgren O Moreau P. International myeloma working group consensus criteria for response and minimal residual disease assessment in multiple myeloma. Lancet Oncol (2016) 17(8):e328–46. doi: 10.1016/S1470-2045(16)30206-6 27511158

[6] Landgren O Rajkumar SV. New developments in diagnosis, prognosis, and assessment of response in multiple myeloma. Clin Cancer Res (2016) 22(22):5428–33. doi: 10.1158/1078-0432.CCR-16-0866 PMC5587183 28151710

[7] Rasche L Chavan SS Stephens OW Patel PH Tytarenko R Ashby C. Spatial genomic heterogeneity in multiple myeloma revealed by multi-region sequencing. Nat Commun (2017) 8(1):268. doi: 10.1038/s41467-017-00296-y 28814763 PMC5559527

[8] Varettoni M Corso A Pica G Mangiacavalli S Pascutto C Lazzarino M. Incidence, presenting features and outcome of extramedullary disease in multiple myeloma: a longitudinal study on 1003 consecutive patients. Ann Oncol (2010) 21(2):325–30. doi: 10.1093/annonc/mdp329 19633044

[9] Pour L Sevcikova S Greslikova H Kupska R Majkova P Zahradova L. Soft-tissue extramedullary multiple myeloma prognosis is significantly worse in comparison to bone-related extramedullary relapse. Haematologica (2014) 99(2):360–4. doi: 10.3324/haematol.2013.094409 PMC3912968 24038024

[10] Cavo M Terpos E Nanni C Moreau P Lentzsch S Zweegman S. Role of ^18^ f-FDG PET/CT in the diagnosis and management of multiple myeloma and other plasma cell disorders: a consensus statement by the international myeloma working group. Lancet Oncol (2017) 18(4):e206–17. doi: 10.1016/S1470-2045(17)30189-4 28368259

[11] Moreau P Attal M Caillot D Macro M Karlin L Garderet L. Prospective evaluation of magnetic resonance imaging and. J Clin Oncol (2017) 35(25):2911–8. doi: 10.1200/JCO.2017.72.2975 PMC5578392 28686535

[12] Rasche L Alapat D Kumar M Gershner G McDonald J Wardell CP. Combination of flow cytometry and functional imaging for monitoring of residual disease in myeloma. Leukemia (2019) 33(7):1713–22. doi: 10.1038/s41375-018-0329-0 PMC6586541 30573775

[13] Biancon G Gimondi S Vendramin A Carniti C Corradini P. Noninvasive molecular monitoring in multiple myeloma patients using cell-free tumor DNA: A pilot study. J Mol Diagn (2018) 20(6):859–70. doi: 10.1016/j.jmoldx.2018.07.006 30165206

[14] Oberle A Brandt A Voigtlaender M Thiele B Radloff J Schulenkorf A. Monitoring multiple myeloma by next-generation sequencing of V(D)J rearrangements from circulating myeloma cells and cell-free myeloma DNA. Haematologica (2017) 102(6):1105–11. doi: 10.3324/haematol.2016.161414 PMC5451343 28183851

[15] Mazzotti C Buisson L Maheo S Perrot A Chretien ML Leleu X. Myeloma MRD by deep sequencing from circulating tumor DNA does not correlate with results obtained in the bone marrow. Blood Adv (2018) 2(21):2811–3. doi: 10.1182/bloodadvances.2018025197 PMC6234381 30355580

[16] Vrabel D Sedlarikova L Besse L Rihova L Bezdekova R Almasi M. Dynamics of tumor-specific cfDNA in response to therapy in multiple myeloma patients. Eur J Haematol (2020) 104(3):190–7. doi: 10.1111/ejh.13358 PMC7065130 31763708

[17] Guo G Raje NS Seifer C Kloeber J Isenhart R Ha G. Genomic discovery and clonal tracking in multiple myeloma by cell-free DNA sequencing. Leukemia (2018) 32(8):1838–41. doi: 10.1038/s41375-018-0115-z PMC6160352 29749395

[18] Mithraprabhu S Morley R Khong T Kalff A Bergin K Hocking J. Monitoring tumour burden and therapeutic response through analysis of circulating tumour DNA and extracellular RNA in multiple myeloma patients. Leukemia (2019) 33(8):2022–33. doi: 10.1038/s41375-019-0469-x 30992504

[19] Kapoor P Kumar SK Dispenzieri A Lacy MQ Buadi F Dingli D. Importance of achieving stringent complete response after autologous stem-cell transplantation in multiple myeloma. J Clin Oncol (2013) 31(36):4529–35. doi: 10.1200/JCO.2013.49.0086 PMC3871514 24248686

[20] Giarin MM Giaccone L Sorasio R Sfiligoi C Amoroso B Cavallo F. Serum free light chain ratio, total kappa/lambda ratio, and immunofixation results are not prognostic factors after stem cell transplantation for newly diagnosed multiple myeloma. Clin Chem (2009) 55(8):1510–6. doi: 10.1373/clinchem.2009.124370 19520760

[21] Ludwig H Milosavljevic D Zojer N Faint JM Bradwell AR Hübl W. Immunoglobulin heavy/light chain ratios improve paraprotein detection and monitoring, identify residual disease and correlate with survival in multiple myeloma patients. Leukemia (2013) 27(1):213–9. doi: 10.1038/leu.2012.197 PMC3542627 22955329

[22] Hari P Pasquini MC Vesole DH. Cure of multiple myeloma – more hype, less reality. Bone Marrow Transplant (2006) 37(1):1–18. doi: 10.1038/sj.bmt.1705194 16258534

[23] Rawstron AC Child JA de Tute RM Davies FE Gregory WM Bell SE. Minimal residual disease assessed by multiparameter flow cytometry in multiple myeloma: impact on outcome in the medical research council myeloma IX study. J Clin Oncol (2013) 31(20):2540–7. doi: 10.1200/JCO.2012.46.2119 23733781

[24] Martinez-Lopez J Lahuerta JJ Pepin F González M Barrio S Ayala R. Prognostic value of deep sequencing method for minimal residual disease detection in multiple myeloma. Blood (2014) 123(20):3073–9. doi: 10.1182/blood-2014-01-550020 PMC4023416 24646471

[25] Lahuerta JJ Paiva B Vidriales MB Cordón L Cedena MT Puig N. Depth of response in multiple myeloma: A pooled analysis of three PETHEMA/GEM clinical trials. J Clin Oncol (2017) 35(25):2900–10. doi: 10.1200/JCO.2016.69.2517 PMC5568033 28498784

[26] Munshi NC Avet-Loiseau H Anderson KC Neri P Paiva B Samur M. A large meta-analysis establishes the role of MRD negativity in long-term survival outcomes in patients with multiple myeloma. Blood Adv (2020) 4(23):5988–99. doi: 10.1182/bloodadvances.2020002827 PMC7724898 33284948

[27] Landgren O Hultcrantz M Diamond B Lesokhin AM Mailankody S Hassoun H. Safety and effectiveness of weekly carfilzomib, lenalidomide, dexamethasone, and daratumumab combination therapy for patients with newly diagnosed multiple myeloma: The MANHATTAN nonrandomized clinical trial. JAMA Oncol (2021) 7(6):862–8. doi: 10.1001/jamaoncol.2021.0611 PMC8050789 33856405

[28] Perrot A Lauwers-Cances V Corre J Robillard N Hulin C Chretien ML. Minimal residual disease negativity using deep sequencing is a major prognostic factor in multiple myeloma. Blood (2018) 132(23):2456–64. doi: 10.1182/blood-2018-06-858613 PMC6284215 30249784

[29] Herve A-L Philippe M Michel A Cyrille H Arnulf B Jill C. Concordance of post-consolidation minimal residualdiseaseratesby multiparametric flow cytometry and next-generation sequencingin CASSIOPEIA. J Clin Oncol (2019) 19(10):E3–E4. doi: 10.1016/j.clml.2019.09.005

[30] Facon T Kumar S Plesner T Orlowski RZ Moreau P Bahlis N. Daratumumab plus lenalidomide and dexamethasone for untreated myeloma. N Engl J Med (2019) 380(22):2104–15. doi: 10.1056/NEJMoa1817249 PMC10045721 31141632

[31] Mateos MV Dimopoulos MA Cavo M Suzuki K Jakubowiak A Knop S. Daratumumab plus bortezomib, melphalan, and prednisone for untreated myeloma. N Engl J Med (2018) 378(6):518–28. doi: 10.1056/NEJMoa1714678 29231133

[32] Cavo M San-Miguel J Usmani SZ Weisel K Dimopoulos MA Avet-Loiseau H. Prognostic value of minimal residual disease negativity in myeloma: combined analysis of POLLUX, CASTOR, ALCYONE, and MAIA. Blood (2022) 139(6):835–44. doi: 10.1182/blood.2021011101 PMC8832474 34289038

[33] Paiva B Puig N Cedena MT Rosiñol L Cordón L Vidriales MB. Measurable residual disease by next-generation flow cytometry in multiple myeloma. J Clin Oncol (2020) 38(8):784–92. doi: 10.1200/JCO.19.01231 31770060

[34] Landgren O Devlin S Boulad M Mailankody S. Role of MRD status in relation to clinical outcomes in newly diagnosed multiple myeloma patients: a meta-analysis. Bone Marrow Transplant (2016) 51(12):1565–8. doi: 10.1038/bmt.2016.222 PMC5571752 27595280

[35] Hervé A-L Valerie L-C Jill C Philippe M Michel A Nikhil M. Minimal residual disease in multiple myeloma: Final analysis of theIFM2009 trial. Blood (2017) 130(Supplement 1, 8):435. doi: 10.1182/blood.V130.Suppl_1.435.435

[36] Cedena MT Martin-Clavero E Wong S Shah N Bahri N Alonso R. The clinical significance of stringent complete response in multiple myeloma is surpassed by minimal residual disease measurements. PLoS One (2020) 15(8):e0237155. doi: 10.1371/journal.pone.0237155 32866200 PMC7458342

[37] Goicoechea I Puig N Cedena MT Burgos L Cordón L Vidriales MB. Deep MRD profiling defines outcome and unveils different modes of treatment resistance in standard- and high-risk myeloma. Blood (2021) 137(1):49–60. doi: 10.1182/blood.2020006731 32693406

[38] Kostopoulos IV Ntanasis-Stathopoulos I Gavriatopoulou M Tsitsilonis OE Terpos E. Minimal residual disease in multiple myeloma: Current landscape and future applications with immunotherapeutic approaches. Front Oncol (2020) 10:860. doi: 10.3389/fonc.2020.00860 32537439 PMC7267070

[39] Flores-Montero J Sanoja-Flores L Paiva B Puig N García-Sánchez O Böttcher S. Next generation flow for highly sensitive and standardized detection of minimal residual disease in multiple myeloma. Leukemia (2017) 31(10):2094–103. doi: 10.1038/leu.2017.29 PMC5629369 28104919

[40] Berger N Kim-Schulze S Parekh S. Minimal residual disease in multiple myeloma: Impact on response assessment, prognosis and tumor heterogeneity. Adv Exp Med Biol (2018) 1100:141–59. doi: 10.1007/978-3-319-97746-1_9 30411265

[41] Dunn-Walters D Townsend C Sinclair E Stewart A. Immunoglobulin gene analysis as a tool for investigating human immune responses. Immunol Rev (2018) 284(1):132–47. doi: 10.1111/imr.12659 PMC6033188 29944755

[42] Sánchez R Ayala R Martínez-López J. Minimal residual disease monitoring with next-generation sequencing methodologies in hematological malignancies. Int J Mol Sci (2019) 20(11):2832. doi: 10.3390/ijms20112832 PMC6600313 31185671

[43] Korde N Roschewski M Zingone A Kwok M Manasanch EE Bhutani M. Treatment with carfilzomib-Lenalidomide-Dexamethasone with lenalidomide extension in patients with smoldering or newly diagnosed multiple myeloma. JAMA Oncol (2015) 1(6):746–54. doi: 10.1001/jamaoncol.2015.2010 PMC6662597 26181891

[44] Van Dongen JJ Langerak AW Brüggemann M Evans PA Hummel M Lavender FL. Design and standardization of PCR primers and protocols for detection of clonal immunoglobulin and T-cell receptor gene recombinations in suspect lymphoproliferations: report of the BIOMED-2 concerted action BMH4-CT98-3936. Leukemia (2003) 17(12):2257–317. doi: 10.1038/sj.leu.2403202 14671650

[45] González D González M Alonso ME López-Pérez R Balanzategui A Chillón MC. Incomplete DJH rearrangements as a novel tumor target for minimal residual disease quantitation in multiple myeloma using real-time PCR. Leukemia (2003) 17(6):1051–7. doi: 10.1038/sj.leu.2402937 12764368

[46] Rustad EH Hultcrantz M Yellapantula VD Akhlaghi T Ho C Arcila ME. Baseline identification of clonal V(D)J sequences for DNA-based minimal residual disease detection in multiple myeloma. PLoS One (2019) 14(3):e0211600. doi: 10.1371/journal.pone.0211600 30901326 PMC6430394

[47] DM A WD J Lofti B Gordon C Merav L Kihyun K. Daratumumab, lenalidomide, anddexamethasone (DRd) versus lenalidomide and dexamethasone (Rd) in relapsed orrefractory multiple myeloma (RRMM): updated efficacy and safety analysis ofpollux. Blood (2017) 130(Supplement 1):739. doi: 10.1182/blood.V130.Suppl_1.739.739

[48] Dimopoulos MA San-Miguel J Belch A White D Benboubker L Cook G. Daratumumab plus lenalidomide and dexamethasone. Haematologica (2018) 103(12):2088–96. doi: 10.3324/haematol.2018.194282 PMC6269302 30237262

[49] Takamatsu H Takezako N Zheng J Moorhead M Carlton VEH Kong KA. Prognostic value of sequencing-based minimal residual disease detection in patients with multiple myeloma who underwent autologous stem-cell transplantation. Ann Oncol (2017) 28(10):2503–10. doi: 10.1093/annonc/mdx340 PMC5834061 28945825

[50] Yao Q Bai Y Orfao A Chim CS. Standardized minimal residual disease detection by next-generation sequencing in multiple myeloma. Front Oncol (2019) 9:449. doi: 10.3389/fonc.2019.00449 31245284 PMC6563351

[51] Medina A Puig N Flores-Montero J Jimenez C Sarasquete ME Garcia-Alvarez M. Comparison of next-generation sequencing (NGS) and next-generation flow (NGF) for minimal residual disease (MRD) assessment in multiple myeloma. Blood Cancer J (2020) 10(10):108. doi: 10.1038/s41408-020-00377-0 33127891 PMC7603393

[52] Ladetto M Brüggemann M Monitillo L Ferrero S Pepin F Drandi D. Next-generation sequencing and real-time quantitative PCR for minimal residual disease detection in b-cell disorders. Leukemia (2014) 28(6):1299–307. doi: 10.1038/leu.2013.375 24342950

[53] Herve A-L Jill C Valerie L-C Marie-Lorraine C Nelly R Xavier L. Evaluation of minimal residual disease (MRD)By next generation sequencing (NGS) is highly predictive of progression FreeSurvival in the IFM/DFCI 2009 trial. (2015) 376(14):1311–1320. doi: 10.1056/NEJMoa1611750

[54] Attal M Lauwers-Cances V Hulin C Leleu X Caillot D Escoffre M. Lenalidomide, bortezomib, and dexamethasone with transplantation for myeloma. N Engl J Med (2017) 376(14):1311–20. doi: 10.1056/NEJMoa1611750 PMC6201242 28379796

[55] Oliva S Genuardi E Belotti A Frascione P Mirko M Galli M. Minimalresidual disease evaluation by multiparameter flowcytometry and next generationsequencing in the forte trial for newly diagnosed multiple myeloma patients. Blood (2019) 394(10192):29–38. doi: 10.1016/S0140-6736(19)31240-1

[56] Moreau P Attal M Hulin C Arnulf B Belhadj K Benboubker L. Bortezomib, thalidomide, and dexamethasone with or without daratumumab before and after autologous stem-cell transplantation for newly diagnosed multiple myeloma (CASSIOPEIA): a randomised, open-label, phase 3 study. Lancet (2019) 394(10192):29–38. doi: 10.1016/S0140-6736(19)31240-1 31171419

[57] Moreau P Hulin C Perrot A Arnulf B Belhadj K Benboubker L. Maintenance with daratumumab or observation following treatment with bortezomib, thalidomide, and dexamethasone with or without daratumumab and autologous stem-cell transplant in patients with newly diagnosed multiple myeloma (CASSIOPEIA): an open-label, randomised, phase 3 trial. Lancet Oncol (2021) 22(10):1378–90. doi: 10.1016/S1470-2045(21)00428-9 34529931

[58] Avet-Loiseau H Sonneveld P Moreau P Offner F Der V Velden V. Daratumumab (DARA) with bortezomib, thalidomide, and dexamethasone (VTd)in transplant-eligible patients (Pts) with newly diagnosed multiple Myeloma(NDMM): Analysis of minimal residual disease (MRD) negativity in CassiopeiaPart 1 and part 2. (2021) 138(Supplement_1):82–3. doi: 10.1182/blood-2021-147897

[59] Oliva S Bruinink DHO Rihova L D'Agostino M Pantani L Capra A. Minimal residual disease assessment by multiparameter flow cytometry in transplant-eligible myeloma in the EMN02/HOVON 95 MM trial. Blood Cancer J (2021) 11(6):106. doi: 10.1038/s41408-021-00498-0 34083504 PMC8175611

[60] Moreau P Zamagni E. MRD in multiple myeloma: more questions than answers? Blood Cancer J (2017) 7(12):639. doi: 10.1038/s41408-017-0028-5 29209089 PMC5802510

[61] Yanamandra U Kumar SK. Minimal residual disease analysis in myeloma - when, why and where. Leuk Lymphoma (2018) 59(8):1772–84. doi: 10.1080/10428194.2017.1386304 PMC6208356 29019452

[62] Oliva S D'Agostino M Boccadoro M Larocca A. Clinical applications and future directions of minimal residual disease testing in multiple myeloma. Front Oncol (2020) 10:1. doi: 10.3389/fonc.2020.00001 32076595 PMC7006453

[63] Anderson KC Auclair D Kelloff GJ Sigman CC Avet-Loiseau H Farrell AT. The role of minimal residual disease testing in myeloma treatment selection and drug development: Current value and future applications. Clin Cancer Res (2017) 23(15):3980–93. doi: 10.1158/1078-0432.CCR-16-2895 28428191

[64] Anderson KC Auclair D Adam SJ Agarwal A Anderson M Avet-Loiseau H. Minimal residual disease in myeloma: Application for clinical care and new drug registration. Clin Cancer Res (2021) 27(19):5195–212. doi: 10.1158/1078-0432.CCR-21-1059 PMC9662886 34321279

[65] Castelli G Pelosi E Testa U. Measurable residual disease in multiple myeloma and in acute myeloid leukemia, an evolving topic. Ann Ist Super Sanita (2021) 57(4):300–13. doi: 10.4415/ANN_21_04_05 35076420

[66] Charalampous C Kourelis T. Minimal residual disease assessment in multiple myeloma patients: Minimal disease with maximal implications. Front Oncol (2021) 11:801851. doi: 10.3389/fonc.2021.801851 35155198 PMC8825476

[67] Ding H Xu J Lin Z Huang J Wang F Yang Y. Minimal residual disease in multiple myeloma: current status. biomark Res (2021) 9(1):75. doi: 10.1186/s40364-021-00328-2 34649622 PMC8515655

[68] Gavriatopoulou M Terpos E Ntanasis-Stathopoulos I Malandrakis P Eleutherakis-Papaiakovou E Papatheodorou A. Consolidation with carfilzomib, lenalidomide, and dexamethasone (KRd) following ASCT results in high rates of minimal residual disease negativity and improves bone metabolism, in the absence of bisphosphonates, among newly diagnosed patients with multiple myeloma. Blood Cancer J (2020) 10(3):25. doi: 10.1038/s41408-020-0297-2 32123158 PMC7052253

[69] McCarthy PL Holstein SA Petrucci MT Richardson PG Hulin C Tosi P. Lenalidomide maintenance after autologous stem-cell transplantation in newly diagnosed multiple myeloma: A meta-analysis. J Clin Oncol (2017) 35(29):3279–89. doi: 10.1200/JCO.2017.72.6679 PMC5652871 28742454

[70] Mateos MV de la Calle VG. Lenalidomide as maintenance for every newly diagnosed patient with multiple myeloma. Lancet Oncol (2019) 20(1):5–6. doi: 10.1016/S1470-2045(18)30764-2 30559052

[71] Dimopoulos MA Moreau P Terpos E Mateos MV Zweegman S Cook G. Corrigendum to 'Multiple myeloma: EHA-ESMO clinical practice guidelines for diagnosis, treatment and follow-up': [Ann oncol 2021; 32(3): 309-322]. Ann Oncol (2022) 33(1):117. doi: 10.1016/j.annonc.2021.10.001 34857439

[72] Goel U Usmani S Kumar S. Current approaches to management of newly diagnosed multiple myeloma. Am J Hematol (2022) 97 (Suppl 1):S3–S25. doi: 10.1002/ajh.26512 35234302

[73] Benboubker L Dimopoulos MA Dispenzieri A Catalano J Belch AR Cavo M. Lenalidomide and dexamethasone in transplant-ineligible patients with myeloma. N Engl J Med (2014) 371(10):906–17. doi: 10.1056/NEJMoa1402551 25184863

[74] Facon T San-Miguel J Dimopoulos MA Mateos MV Cavo M van Beekhuizen S. Treatment regimens for transplant-ineligible patients with newly diagnosed multiple myeloma: A systematic literature review and network meta-analysis. Adv Ther (2022). doi: 10.1007/s12325-022-02083-8 PMC9056460 35246820

[75] Hansen MH Cédile O Larsen TS Abildgaard N Nyvold CG. Perspective: sensitive detection of residual lymphoproliferative disease by NGS and clonal rearrangements-how low can you go? Exp Hematol (2021) 98:14–24. doi: 10.1016/j.exphem.2021.03.005 33823225

[76] Rustad EH Boyle EM. Monitoring minimal residual disease in the bone marrow using next generation sequencing. Best Pract Res Clin Haematol (2020) 33(1):101149. doi: 10.1016/j.beha.2020.101149 32139014 PMC7133460

